# Adverse events and survival after closing- and opening-wedge high tibial osteotomy: a comparative study of 412 patients

**DOI:** 10.1007/s00167-015-3644-2

**Published:** 2015-05-31

**Authors:** T. Duivenvoorden, P. van Diggele, M. Reijman, P. K. Bos, J. van Egmond, S. M. A. Bierma-Zeinstra, J. A. N. Verhaar

**Affiliations:** 1 Department of Orthopaedics, Erasmus University Medical Centre, PO Box 2040, 3000 CA Rotterdam, The Netherlands; 2 Department of General Practice, Erasmus University Medical Centre, Rotterdam, The Netherlands

**Keywords:** Opening-wedge high tibial osteotomy, Closing-wedge high tibial osteotomy, Adverse events, Survival

## Abstract

**Purpose:**

Varus medial knee osteoarthritis (OA) can be treated with a closing-wedge (CW) or opening-wedge (OW) high tibial osteotomy (HTO). Little is known about the adverse event (AE) rate of these techniques. The purpose of this study was to examine the AE rate and survival rate of a consecutive series of 412 patients undergoing CW- or OW-HTO.

**Methods:**

Medical records were retrospectively screened, and all patients who underwent HTO from 1993 to 2012 at the Erasmus University Medical Centre were assessed with a self-administered questionnaire. Patients filled in the intermittent and constant osteoarthritis pain score, knee injury and osteoarthritis outcome score, and a general questionnaire focusing on AE.

**Results:**

Medical records of 412 patients (354 CW- and 112 OW-HTOs) were screened. Of the 358 eligible patients, 291 (81 %) returned their questionnaire. A total of 80 AE (17 %) were found in 466 osteotomies. In the CW-group, 47 (13 %) serious adverse events (SAE) and 2 (0.6 %) AE were found. In the OW-group, 17 (15 %) SAE and 14 (13 %) AE were found. The most common AE was in 14 (4 %) patients of the CW-group sensory palsy of the common peroneal nerve. The most common AE in the OW-group was persistent pain at the iliac crest [11 (9.8 %) patients]. Hardware was removed in 48 % of the CW-osteotomies and 71 % of the OW-osteotomies (*p* < 0.05). The probability of survival was 75 % after 10 years in the CW-group versus 90 % in the OW-group (*p* < 0.05). In both groups, an equal number of patients were “in need for prosthesis” according to OARSI criteria.

**Conclusion:**

OW-HTO was associated with more AE than CW-HTO. OW-HTO resulted in better survival than CW-HTO. However, in both groups an equal number of patients were in need for prosthesis.

**Level of evidence:**

Retrospective comparative study, Level III.

## Introduction

Knee osteoarthritis (OA) is one of the most common joint disorders and causes considerable pain and immobility. In case of varus alignment, the medial compartment is mostly affected [1, 17]. Varus medial knee OA in young and active patients can be treated with a valgus high tibial osteotomy (HTO). Various techniques are available among which closing-wedge (CW) and opening-wedge (OW) HTO are performed most frequently. Both have advantages and disadvantages, and overall good short- and midterm outcomes have been reported [2, 5, 7, 12, 14, 21].

Although types of adverse events after HTO are well described, little is known about their actual incidence [3, 5, 15, 18, 19]. In a prospective study of 40 patients, Van Bekerom et al. found significant more adverse events in an OW-group, whereas in a randomized controlled trial of 50 patients, Gaasbeek et al. found a higher adverse event rate in a CW-group. Song et al. found no difference in adverse events rate in their retrospective study of 194 patients [8, 18, 19]. Thus, only a few relatively small studies have compared the adverse event rate of CW- and OW-HTO, and the results of these studies are contradictory.

The success of a HTO is expressed in the number of years until conversion to a joint prosthesis is performed. Several studies have studied the survival of the HTO and factors influencing the survival [6, 12, 21]. They all defined failure as redo procedure of the HTO or conversion to unicompartmental knee arthroplasty (UKA) or total knee arthroplasty (TKA). However, this end-point may introduce a decision bias and thus lead to an overestimation of the survival, because the decision to convert an HTO is affected by the opinion of patient as well as surgeon. Patients who do not undergo further surgery do not necessarily have a good result and might have high pain scores and a low functional outcome. Therefore, the Osteoarthritis Research Society International (OARSI) defined criteria for a surrogate measure of the “need for joint replacement surgery” [9]. With these criteria, it is possible to define a non-survivor based on the pain score and functional outcome. Adjustment of the original survival rate with this patient-reported outcome-based measure would result in a more accurate estimate of the real survival.

The hypothesis of this retrospective study was to find a higher adverse event rate and lower survival rate in patients undergoing OW-HTO in comparison with CW-HTO.

## Materials and methods

The medical records of all patients who underwent CW- or OW-HTO at the [Erasmus Medical Centre] (a university teaching hospital) between 1993 and 2012 were screened. This period was chosen for the reason that medical records are preserved for at least 20 years in our hospital and we would achieve a minimal follow-up of 1 year.

All 412 patients were asked to fill in a written questionnaire in 2013. When patients did not respond within 3 weeks, they were contacted by telephone. When these patients did not answer their telephone, a reminder was sent by mail after checking their address in the municipal administration.

### Measurements

Patient characteristics such as gender, preoperative age, body mass index (BMI) and hip–knee–ankle angle (HKA angle) were collected for all 412 patients. Medical records were screened to identify the osteotomy technique, operating time, type of fixation material and adverse events. Pain, functional status and adverse events were measured with patient-reported outcome measures (PROMs). Pain and functional status were measured to define a survivor according to the OARSI criteria [9].

Pain was measured with the intermittent and constant osteoarthritis pain (ICOAP) score (0–100) [10]. The functional status was measured with the Knee injury and Osteoarthritis Outcome Score–Physical Function Short Form (KOOS-PS) [3, 4, 16]. The KOOS-PS is intended to elicit people’s opinions about the difficulties they experience with activity due to problems with their knee. Standardized response options are given (5-point Likert scale), and each question was scored from 0 to 4. Then, a normalized score from 0 to 100 is calculated. The criteria defined by the OARSI to determine patients in need for joint replacement surgery were used. They concluded that the sum score [pain (measured with the ICOAP (0–100)) + physical function (measured with the KOOS-PS (0–100)) >80] is a discriminatory cut-off point to define an indication for joint replacement [9].

### Adverse events

Adverse events were initially assessed from medical records. Moreover, to avert under-registration, adverse events were also assessed with a self-administered questionnaire at follow-up. Patients were specifically asked in this questionnaire for wound infection, thromboembolism, bleeding, paraesthesia, dropping foot, reflex sympathetic dystrophy syndrome, persistent pain or pain at the iliac crest, non-union. When patients scored positive for an adverse event, the adverse event was checked by a telephonic examination. In this study, the adverse events of all 412 patients are presented, of whom the medical records were screened.

Adverse events were classified into adverse events (AE) and serious adverse events (SAE). Adverse events were defined as serious when patients have an undesirable experience associated with a medical intervention leading to death, a life-threatening situation, initial or prolonged hospitalization, disability or permanent damage, or a needed intervention to prevent permanent impairment or damage according to the definition of the FDA.

### Surgical techniques

#### Closing-wedge group

CW-HTO was performed with a lateral approach. The common peroneal nerve (CPN) was exposed and retracted. The proximal tibiofibular joint was opened, and 1 cm of the proximal tibiofibular bone was resected. The proximal osteotomy site and the slope of the osteotomy were marked using two Kirschner (K)-wires. Under C-arm guidance, the first osteotomy was performed with an oscillating saw and completed with an osteotome. The second osteotomy was performed with help of an aiming device (Arthrex, Naples, Florida). Fixation was achieved using two staples or a Tomofix plate. Different types of fixation material were used over years; staples (Stryker, Schönkirchen, Germany), Tomofix plate (Synthes GmbH, Oberdorf, Switzerland) and Puddu plate (Arthrex, Naples, Florida). Before closing the wound, a fasciotomy of the anterior compartment was performed.

#### Opening-wedge group

OW-HTO was performed with an anteromedial incision. The pes anserinus tendons and medial collateral ligament were dorsally retracted. Under C-arm guidance, two K-wires were placed in an oblique fashion from medial to lateral to serve as a cutting guide for the osteotomy. An osteotomy was performed using an oscillating saw. Care was taken to avoid violation of the lateral cortex. The distracting osteotomes were placed ventrally and dorsally and gradually distracted to achieve the right alignment. OW-osteotomies were fixated using either a Puddu plate without plate locking screws (until 2006) or Tomofix plate (from 2006 until now). In 56 patients with large wedges of the OW-group, a spongioplasty was performed using autologous bone harvested at the iliac crest. The Ethics Committee of the Erasmus Medical Centre approved the protocol (MEC-2013-140).

### Statistical analysis

Statistical analysis was performed using PASW statistics version 20 (SPSS science Inc., Chicago, USA), and a *p* value <0.05 was considered as statistically significant. Data of CW- and OW-HTO patients are presented separately. Between-group differences were tested with the independent *t* test (Student’s *t* test) or *χ* ^2^ test.

To evaluate the presence of a possible selective dropout during follow-up, baseline characteristics of the responders (those who filled in the questionnaire) were compared with the non-responders using the Kruskal–Wallis or *χ* ^2^ test.

Differences between CW- and OW-HTO patients were analysed using the independent *t* test (Student’s *t* test) or *χ* ^2^ test.

Multiple survival analyses according to Kaplan and Meier were carried out. In the first survival analysis, conversion to a UKA or TKA was considered as end-point. In the second survival analysis, “being in need for a UKA or TKA” according to the OARSI criteria was considered as end-point [13]. Patients with a conversion to a UKA or TKA were also considered as “being in need for a UKA or TKA”. Differences in survival between CW- and OW-HTO were calculated using the log rank test (Table 1).

**Table 1:** Patient characteristics of the total study population, separately for opening-wedge versus closing-wedge HTO and responders versus non-responders

	Total group of osteotomies (*n* = 466)	Closing-wedge osteotomy (*n* = 354)	Opening-wedge osteotomy (*n* = 112)	Responders (*n* = 291)	Non-responders (*n* = 121)^a^
Follow-up time (years)	9.8 (4.9)	10.6 (5.1)^†^	7.4 (3.2)^†^	NA	NA
Women, *n* (%)	190 (40.8)	151 (42.7)	39 (34.8)	134 (46)	36 (30)
Age (yrs)^b^	49.2 (9.3)	49.4 (9.0)	48.7 (10.1)	49.7 (8.7)	47.9 (10.6)
BMI (kg/m^2^)^b^	29.1 (5.4)	29.5 (5.8)	28.5 (4.5)	29.0 (5.4)	29.3 (5.3)
HKA angle (°)^b,c^	6.6 (2.6)	6.3 (2.2)^†^	7.4 (3.5)^†^	6.6 (2.6)	6.6 (2.7)
Surgery time (min)	116.9 (30.3)	112.7 (28.8)^†^	130.3 (31.4)^†^	118.8 (30.7)	112.2 (29.1)
Duration of hospitalization (days)	5.5 (2.9)	5.5 (2.3)	5.5 (4.2)	5.2 (2.4)	6.1 (3.6)

All values are presented as mean (±SD) unless stated otherwise

*BMI* body mass index, *deg* degree, *HTO* high tibial osteotomy, *HKA* angle hip–knee–ankle angle, *min* minutes, *NA* not applicable, *yrs* years

^†^ *p* < 0.05 for difference between the two groups

^a^77 % of the lost patients underwent closing-wedge HTO

^b^The preoperative values are presented

^c^A positive value means varus malalignment

**Fig. 1:**
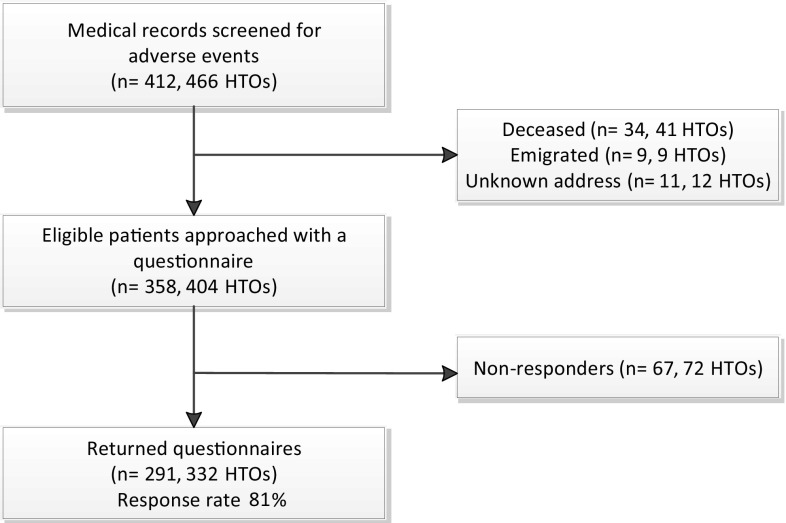
Flowchart of the study. *HTO* high tibial osteotomy

We have found in this study a RR of 2.0 with 49/354 AE in the CW-group and 31/112 AE in the OW-group. The power of this study with 354 patients in the CW-group and 112 patients in the OW-group was 0.94 to find a RR of 2.0. Concerning the survival after HTO, we found in this study a RR of 3.0 with 73/354 non-survivors in the CW-group and 8/112 in the OW-group. The power of this study with 354 patients in the CW-group and 112 patients in the OW-group was 0.99 to find a RR of 3.0 (Fig. 1).

**Fig. 2:**
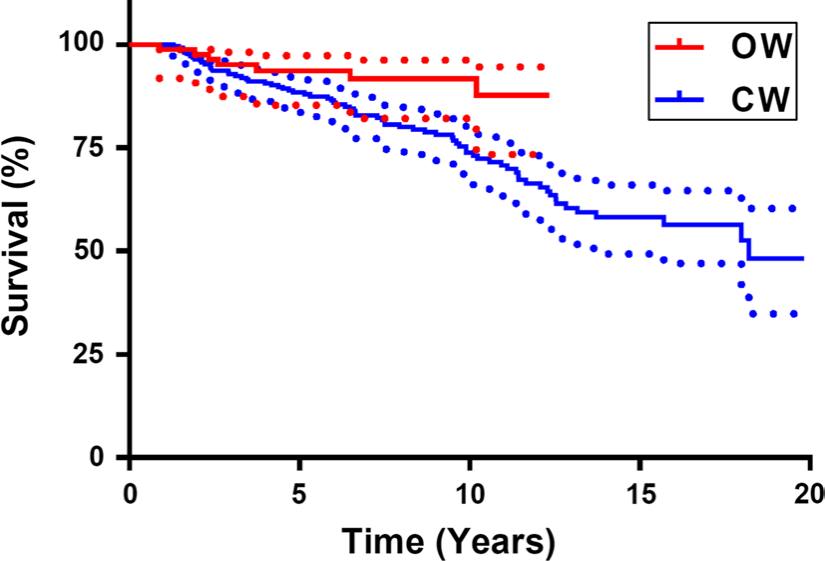
Survival curve of closing- and opening-wedge osteotomy. Survival considered with conversion to UKA or TKA as end-point

## Results

### Adverse events

A total of 80 AE (17 %) were found in 466 osteotomies. In the CW-group, 47 (13 %) SAE and 2 (0.6 %) AE were found. In the OW-group, 17 (15 %) SAE and 14 (13 %) AE were found. The most common AE was in 14 (4 %) patients of the CW-group temporary sensory palsy of the CPN. In the OW-group, the most common AE persistent pain was in 11 (19.7 %) patients at the iliac crest of those who had cancellous bone grafting from the iliac crest in OW-HTO. Moreover, a non-union occurred in 12 patients (8 (2.3 %) in the CW-group and 4 (3.6 %) in the OW-group). Hardware was removed in 48 % of the CW-osteotomies and 71 % of the OW-osteotomies (*p* < 0.05). All adverse events are outlined in Table 2.

**Table 2:** Adverse events for the closing- and opening-wedge group

	Number of events, *n* (*%*)	
	Closing-wedge osteotomy (*n* = 354)	Opening-wedge osteotomy (*n* = 112)
Serious adverse events		
Sensory palsy of the CPN	14 (4.0)	0
Motor palsy of the CPN	1 (0.3)	0
Pseudoarthrosis	8 (2.3)	4 (3.6)
Wound infection treated with antibiotics	6 (1.7)	5 (4.5)
Fracture of the tibial plateau	2 (0.6)	2 (1.9)
Re-HTO^a^	7 (2.0)	3 (2.7)
Delayed union	1 (0.3)	0
Lesion of the ATA	1 (0.3)	0
Malposition of hardware	1 (0.3)	0
Deep venous thrombosis	2 (0.6)	0
Pulmonary embolus	0	1 (0.9)
Infection of the urinary tract	2 (0.6)	1 (0.9)
Post-surgery diffuse lung emphysema	1 (0.3)	0
Compartment syndrome	1 (0.3)	1 (0.9)
Hardware removal^b^	169 (47.7)	79 (70.5)
Adverse events		
Iliac crest pain	0	11 (19.7)^c^
Wound infection without antibiotic treatment	1 (0.3)	2 (1.9)
CRPS	1 (0.3)	1 (0.9)

One hundred and twenty patients did not return their questionnaire for several reasons; their adverse events were only assessed by medical record screening

*CPN* common peroneal nerve, *ATA* anterior tibial artery, *CRPS* complex regional pain syndrome

^a^Re-HTO was performed because of overcorrection or undercorrection or loss of correction

^b^Ten hardware removals in the closing-wedge group and two in the opening-wedge group were performed prior to total knee arthroplasty

^c^Fifty-six patients (50 %) of the opening-wedge group underwent spongioplasty with autologous bone harvested at the iliac crest. Of these patients, 11 patients reported pain at the iliac crest for more than 6 weeks

### Survival

**Fig. 3:**
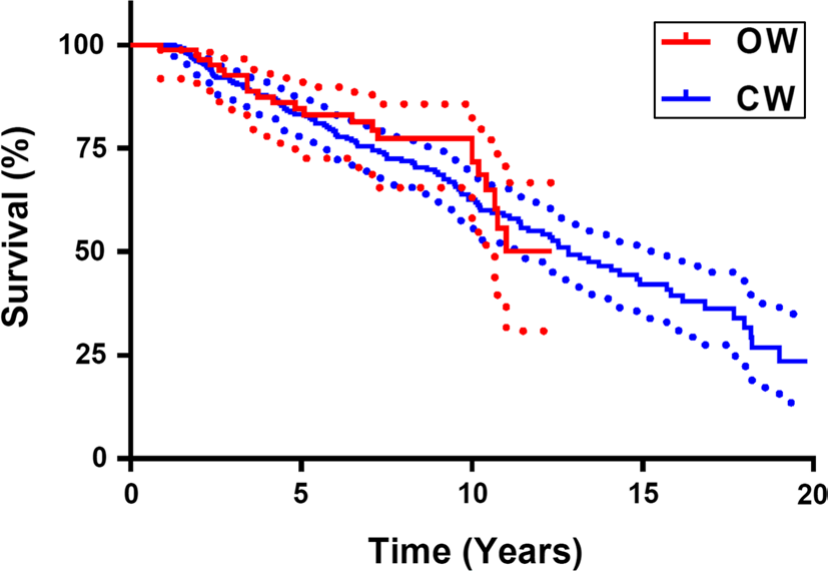
Survival curve of closing- and opening-wedge osteotomy. Survival considered with “being in need for a UKA of TKA” according to the OARSI criteria in addition to “being conversed to UKA or TKA” as end-point

During the follow-up period, 81 osteotomies (17.4 %) have been revised to UKA or TKA: 73 in the CW-group and eight in the OW-group (Fig. 3). When conversion to UKA or TKA was considered as end-point, the OW-group had a better survival than the CW-group (*p* < 0.05; Fig. 2). When the OARSI criteria for “being in need for a UKA or TKA” was considered as end-point and this was added to conversion to a prosthesis, no difference in survival between CW- and the OW-group was found.

## Discussion

The aim of this study was to present the adverse event rate and survival rate of 466 HTOs performed in our university teaching hospital. The most important finding of this study was the overall adverse event rate of 28 % in the OW-group and 14 % in the CW-group. This AE rate is lower than reported in several studies. Other studies show adverse event rates ranging from 24 to 63 % [2, 13, 18, 19]. However, Gaasbeek et al. reported an adverse event rate of 12 % [8]. The higher adverse event rate in the OW-group is in agreement with Van Bekerom et al. who reported 55 % adverse events in the OW-group (*n* = 20) and 20 % in the CW-group (*n* = 20). However, Song et al. found more adverse events in the CW-group (28 %, *n* = 104) than in the OW-group (20 %, *n* = 90). Miller et al. and Floerkemeier et al. studied only adverse events after OW-osteotomy. They reported adverse event rates of 37 % (*n* = 46) and 6 % (*n* = 533), respectively [7, 15]. Results of the different studies seem to be contradictory. In the majority of the studies, only medical records were screened to identify adverse events. This could have led to an underestimation. The strength of this study is the additional information assessed with a self-administered questionnaire after screening of the medical records in a relatively large number of patients. Consequently, we assume that our results are more accurate estimate of the real adverse event rate.

Different types of fixation material were used over years; however, no difference was found in removal rate between staples and plates. The hardware removal rates are similar to those in the literature. Multiple studies report a hardware removal rate of >50 % of the patients in an OW-group and significantly more hardware removals in the OW-group in comparison with the CW-group [5, 11, 20]. The more superficial medial position of hardware with less coverage of soft tissue might be a possible explanation for the higher hardware removal rate in the OW-group.

Another major adverse event was pain at the iliac crest, caused by harvesting the cancellous bone for gap filling in an OW-HTO. In a recent randomized controlled trial, Zorzi and colleagues concluded that a cancellous bone graft in wedges <12.5 mm is not necessary [22]. It is clear that the complication rate will decrease in the OW-group when bone grafting can be avoided.

During the follow-up period, 17.4 % of the osteotomies have been revised to UKA or TKA. When conversion to prosthesis was taken as end-point, the OW-group had a significantly better survival than the CW-group. For CW-osteotomy, the probability of survival was 75 % after 10 years and 50 % after 20 years. For OW-osteotomy, the probability of survival was 90 % after 10 years. Hui et al. found in their retrospective study of 455 CW-osteotomies a probability of survival of 79 % after 10 years and 56 % at 15 years, which is comparable with our results [14].

When “being in need for a UKA or TKA” according to the OARSI criteria in addition to joint replacement [9] was considered as end-point, no difference in survival between the CW- and the OW-groups was found. So it seems that patients with a CW-HTO were converted earlier than patients with an OW-HTO. There is no existing literature to compare this result.

Some limitations of our study need to be addressed. Adverse events were measured retrospectively with medical record screening. This could lead to an underestimation of the adverse event rate, due to known under-reporting in medical records. For this reason, we have also assessed the adverse events with a self-administered questionnaire. We identified with this additional information more cases of wound infection, deep venous thrombosis, iliac crest pain and sensory palsy of the CPN. The response rate of the questionnaire was 81 %, which is high for a study with patients with a follow-up until 20 years. So the results of this study could still be an underestimation of the real adverse event rate; particularly, the number of wound infections, deep venous thrombosis, iliac crest pain and cases of sensory palsy of the CPN could be underestimated. Because of recall bias, all minor adverse events may be under-represented. However, to the best of our knowledge this is the first study in a large group of patients with this approach.

Secondly, the HTO procedures were performed and supervised by different surgeons over the study period in a university teaching hospital. Moreover, during the study period, the OW-technique was introduced. Although all surgeons were experienced, a single surgeon with experience in both techniques would have been preferable to reduce possible operator-dependent variability. Introduction of a new operation technique could lead to an increased risk of adverse events. However, this situation reflects common orthopaedic practice and improves the generalizability of the results.

## Conclusion

OW-HTO was associated with more adverse events than CW-HTO. Hardware was removed more often in patients with CW- than OW-HTO. OW-HTO resulted in a better survival than CW-HTO; however, an equal proportion of patients were in need for prosthesis in both groups.
